# Competitive Binding of Gingerol Homologs to Fish Myofibrillar Protein Reduces the Retention of Aldehydic Off-Odor Compounds

**DOI:** 10.3390/foods15183216

**Published:** 2026-09-11

**Authors:** Hui-Lin Zhao, Yu-Ting Jiao, Lei Qin, Jia-Nan Chen, Xu-Hui Huang

**Affiliations:** 1 State Key Laboratory of Marine Food Processing and Safety Control, National Engineering Research Center of Seafood, Collaborative Innovation Center of Seafood Deep Processing, Dalian Technology Innovation Center for Chinese Prepared Food, School of Food Science and Technology, Dalian Polytechnic University, Dalian 116034, China; 2 College of Light Industry, Liaoning University, Shenyang 110036, China

**Keywords:** mackerel, myofibrillar protein, gingerol, aldehyde, flavor binding, HS-SPME–GC–MS, molecular docking, molecular dynamics

## Abstract

Persistent aldehydic off-notes in fish products are influenced not only by lipid oxidation but also by protein-associated retention of odor-active aldehydes. A remaining question is how gingerol side-chain length and aldehyde structure jointly alter this apparent retention. Mackerel myofibrillar protein (MP) was therefore combined with (E)-2-decenal (T2D), (E,E)-2,4-decadienal (DDE), or trans-4,5-epoxy-(E)-2-decenal (E2D), with or without 6-, 8-, or 10-gingerol. Headspace solid-phase microextraction–gas chromatography–mass spectrometry, interaction disruptors, spectroscopy, molecular docking, and 100 ns molecular dynamics simulations were integrated. All three gingerols decreased the apparent aldehyde-binding ratio of MP. At 125 µmol/g protein, 10-gingerol reduced T2D, DDE, and E2D binding from approximately 81%, 72%, and 85% to 68%, 65%, and 68%, respectively. Spectroscopic responses were consistent with changes in the optical and conformational environment; fluorescence was interpreted as apparent quenching without numerical inner-filter correction, and CD band changes provided evidence of a treatment-related conformational response without assigning exact secondary-structure fractions. Docking ranked 8-gingerol most favorably, whereas the selected binary MYH7-10-gingerol trajectory showed greater pocket residence and a more favorable MM/GBSA estimate than the corresponding MYH7-DDE trajectory. The combined evidence supports a three-layer working model involving matrix partitioning, protein-level site accessibility, and pocket-level competition. These findings provide a mechanistic basis for sensory validation rather than direct proof of deodorization.

## 1. Introduction

Aldehydic off-notes remain a practical obstacle to the consistent sensory quality of processed fish. In lipid-rich species such as mackerel, unsaturated fatty-acid oxidation generates odor-active aldehydes, while matrix chemistry influences both their formation and their subsequent distribution among water, lipids, headspace, and muscle proteins [1,2,3,4]. This study therefore focuses on three structurally distinct C10 aldehydes—(E)-2-decenal (T2D), (E,E)-2,4-decadienal (DDE), and trans-4,5-epoxy-(E)-2-decenal (E2D)—whose low odor thresholds and continued oxidation make their protein-associated retention relevant even at modest bulk concentrations [2,4,5,6].

T2D is associated with fatty or green notes that can become oxidized as concentration and matrix change; DDE can contribute fatty or deep-fried aromas at low levels but oxidized or painty notes during fish-oil deterioration; and E2D has a metallic/fatty character and an exceptionally low reported air threshold [2,3,4,5,6,7]. Across muscle-food matrices, processing and matrix constituents can redirect precursor oxidation and thereby alter aldehyde generation [8,9], whereas the sensory expression of these C10 aldehydes also depends on concentration, co-occurring volatiles, and partitioning [4,7,10,11]. Protein-associated retention is therefore relevant to the persistence and later release of these odorants, whereas the headspace-accessible fraction contributes to their immediate sensory expression. A lower retention metric alone does not establish a lower perceived off-odor intensity; the fate of the released aldehydes must also be considered.

Myofibrillar proteins (MPs) are major structural components of fish muscle and provide hydrophobic cavities, polar groups, and reactive residues for volatile binding. Wang et al. (2024) reported that low-dose free-radical oxidation changed surface hydrophobicity, tryptophan fluorescence, and secondary structure in chicken MP [12]. Early work showed that lipid-derived aldehydes can modify fish myosin, while later headspace and molecular studies demonstrated that MP retention depends on protein concentration, ionic strength, pH, aldehyde structure, and protein conformation [13,14]. The myosin head has been identified as a principal off-odor-binding region [15], and integrated spectroscopy–simulation studies have distinguished reversible physicochemical association—often dominated by hydrophobic contacts—from possible irreversible covalent reactions [16,17,18]. These findings make MP a plausible control point, but they also show why a single binding constant or a single spectroscopic signal cannot describe the whole process.

Phenolic compounds offer one route to modify this control point. In silver-carp MP, rosmarinic acid, carnosic acid, and carnosol formed phenolic–protein complexes, reduced available hydrophobic sites, and promoted the release of retained fishy compounds [19]. Rosemary extracts and selected phenolics have also reduced fishy odor or altered protein-bound odorants in fish systems [20,21,22]. More recently, spice-derived compounds were shown to change MP conformation and its aldehyde-binding capacity [23]. Gingerols are relevant within this class because 6-, 8-, and 10-gingerol share a hydroxyl–methoxy phenyl group but differ in aliphatic side-chain length. Their binding to serum albumin is hydrophobically sensitive [24], and 6-gingerol can alter MP structure under heating or through non-covalent association [25,26]. Comparisons across gingerol homologues and proteins further suggest that longer chains can increase hydrophobic association, although the preferred homologue remains receptor- and pocket-dependent [27]. Mechanistically, the shared oxygen-containing groups provide potential polar contacts, while the differing aliphatic chains may alter hydrophobic association and access to protein binding regions. Gingerol association may thus limit aldehyde retention through overlapping site occupancy and changes in protein conformation, alongside changes in matrix partitioning [19,23,24]. From a processing perspective, gingerol addition during curing may reduce the protein-associated retention of aldehydes and facilitate their subsequent release or removal, thereby providing a potential strategy for limiting the persistence of aldehydic off-notes in cured fish products. However, how this effect varies with gingerol side-chain length and aldehyde structure remains unclear.

The key gap is therefore not whether phenolics interact with proteins, but how three homologous gingerols jointly influence matrix partitioning, MP conformation, and competition for a selected myosin-head pocket when the aldehydes have the same carbon number but different unsaturation or epoxidation. This study addressed that gap using mackerel MP, 6-, 8-, and 10-gingerol, and three C10 aldehydes: (E)-2-decenal (T2D), (E,E)-2,4-decadienal (DDE), and E2D. We tested the working hypothesis that increasing gingerol hydrophobicity would generally reduce apparent MP retention, while aldehyde structure and pocket complementarity would prevent a strictly monotonic response across all measurements. Headspace quantification was combined with interaction perturbation, UV–visible absorption, intrinsic and synchronous fluorescence, circular dichroism (CD), docking, and 100 ns molecular dynamics (MD). The intended contribution is an integrated, testable explanation of where the observed effect arises, rather than a claim that one assay alone proves direct displacement.

## 2. Materials and Methods

### 2.1. Materials and Reagents

Fresh Spanish mackerel (*Scomberomorus niphonius*) was purchased from Qianhe Market (Dalian, China). Dorsal muscle was collected after removal of skin, bone, and viscera. (E)-2-Decenal (T2D), (E,E)-2,4-decadienal (DDE), trans-4,5-epoxy-(E)-2-decenal (E2D), 6-gingerol, 8-gingerol, and 10-gingerol were obtained from Aladdin Biochemical Technology Co., Ltd. (Shanghai, China). The stated purity of each aldehyde standard was 99.9%; gingerols were used as supplied. Ethanol, NaCl, KCl, phosphate salts, Na_2_SO_4_, urea, guanidine hydrochloride (GuHCl), propylene glycol (PG), and other reagents were of analytical or chromatographic grade.

### 2.2. Extraction of Myofibrillar Protein

MP was isolated under chilled conditions using a procedure adapted for fish muscle [28]. All extraction solutions were pre-cooled at 4 °C overnight. Fresh dorsal muscle was minced, weighed, and mixed with four volumes (1:4, *w*/*v*) of 10 mmol/L potassium phosphate buffer (pH 7.0) containing 0.1 mol/L KCl. The suspension was homogenized using a T18 homogenizer (IKA, Staufen, Germany) in an ice bath at 1000 rpm for 30 s, rested for 30 s, and subjected to two additional homogenization cycles. It was then centrifuged using a CF16RN refrigerated centrifuge (Hunan Xiangyi Laboratory Instrument Development Co., Ltd., Changsha, China) at 8000× *g* for 10–15 min at 4 °C, and the supernatant was discarded. The pellet was resuspended in four volumes of the same phosphate–KCl buffer, homogenized, and centrifuged twice more, giving three phosphate-buffer washes in total; the pellet was retained after each wash. The resulting pellet was subsequently washed three times with four volumes of 0.1 mol/L NaCl using the same homogenization cycle, followed each time by centrifugation at 8000× *g* for 15 min at 4 °C and retention of the pellet. After the final NaCl wash, the protein fraction was passed through three layers of gauze when required by sample consistency and weighed. It was then mixed with four volumes (1:4, *w*/*v*) of 20 mmol/L potassium phosphate buffer (pH 7.0) containing 0.6 mol/L NaCl and stirred for 60 min in an ice bath. The suspension was centrifuged at 8000 rpm for 15–20 min at 4 °C, and the supernatant was collected as the MP solution. Protein concentration was determined by the bicinchoninic acid assay and adjusted to 6 mg/mL.

### 2.3. Preparation of Gingerol, Aldehyde, and MP Systems

Individual gingerol and aldehyde stock solutions (1 mg/mL) were prepared in ethanol, diluted to 0.1 mg/mL, protected from light, and kept cold until use. For each experiment, MP (6 mg/mL) was combined with one aldehyde at a final concentration of 10 mg/L. Gingerol-containing groups received 6-, 8-, or 10-gingerol at 125 μmol/g protein. Mixtures were sealed in 20 mL headspace vials and incubated for 12 h at 4 °C before analysis. Unless otherwise stated, an MP–aldehyde sample without gingerol served as the comparison group.

### 2.4. HS-SPME–GC–MS Quantification and Apparent Binding Ratio

Aldehydes were quantified using calibration curves based on aldehyde-to-cyclohexanone peak-area ratios, with cyclohexanone serving as the internal standard. Sealed vials were equilibrated at 60 °C for 10 min, after which a 50/30 μm divinylbenzene/carboxen/polydimethylsiloxane fiber (2 cm) was exposed to the headspace for 15 min. The fiber was desorbed at 250 °C for 5 min in an Agilent 7890B gas chromatograph coupled to an Agilent 7010B triple-quadrupole mass spectrometer (Agilent Technologies, Santa Clara, CA, USA) equipped with an HP-5 column (30 m × 0.25 mm × 0.25 μm; Agilent Technologies, Santa Clara, CA, USA). The oven temperature was initially 45 °C, increased at 15 °C/min for 3 min, then at 5 °C/min for 10 min, and finally at 15 °C/min for 5 min. For each aldehyde, calibration standards were measured at five concentration levels (0.25, 0.50, 1.00, 2.00, and 3.00 μg/mL), covering a range of 0.25–3.00 μg/mL. Each concentration level was analyzed in triplicate. For each calibration, the aldehyde concentration (μg/mL) was used as the x variable and the dimensionless peak-area ratio A_aldehyde/A_cyclohexanone as the y variable. The linear regressions were as follows: T2D, y = 0.3301x − 0.0058 (R^2^ = 0.9995); DDE, y = 0.1208x − 0.0088 (R^2^ = 0.9988); and E2D, y = 0.0436x − 0.0015 (R^2^ = 0.9986). These R^2^ values describe the fit of the reported calibration data; they do not, by themselves, exclude systematic residual patterns or establish quantitative accuracy throughout the calibration range. Internal-standard correction and matrix-sensitive calibration procedures can reduce bias in headspace volatile quantification [29,30].

The apparent binding ratio was calculated as B_app (%) = (C_air − C_MP)/C_air × 100, where C_air was the aldehyde concentration calculated for the matched air-only blank vial without MP or liquid matrix and C_MP was the concentration calculated after incubation with MP. Because HS-SPME measures an equilibrium-dependent headspace signal and the reference was an air blank, B_app is reported as an operational headspace-derived metric rather than an absolute molecular binding fraction.

### 2.5. Interaction-Disruptor Experiments

To probe the apparent contribution of non-covalent interactions, Na_2_SO_4_, urea, GuHCl, or PG was added to MP–aldehyde–gingerol mixtures to final concentrations of 0.5 mol/L, 5 mol/L, 1 mol/L, or 20% (*v*/*v*), respectively. The remaining steps followed Section 2.4. Results were expressed relative to the matched sample without disruptor. Urea and GuHCl can induce different unfolding and aggregation pathways rather than selectively eliminating a single bond type [31]; accordingly, these treatments were interpreted as system perturbations, not as one-to-one chemical-bond tests.

### 2.6. UV–Visible and Fluorescence Spectroscopy

UV-visible spectra were recorded from 200 to 420 nm with a UV-2100B UV-visible spectrophotometer (Shanghai Metash Instruments Co., Ltd., Shanghai, China) using quartz cells and the corresponding buffer as the baseline. For intrinsic fluorescence measurements, MP–aldehyde mixtures were incubated for 40 min at 298 K, and emission spectra were recorded using a F-2700 fluorescence spectrophotometer (Hitachi High-Tech Corporation, Tokyo, Japan). Excitation was set at 280 nm; emission was collected from 300 to 450 nm with 5 nm excitation and emission slit widths. Synchronous fluorescence was obtained at fixed wavelength intervals (Δλ = λem − λex) of 15 and 60 nm, used as Tyr- and Trp-weighted responses, respectively. No numerical inner-filter correction was applied in the present analysis. Because a defensible correction requires matched absorbance values at the excitation and emission wavelengths for each diluted fluorescence sample, the measured intensities were interpreted only as apparent quenching, and no fluorescence-derived binding constants were calculated [32].

### 2.7. Circular Dichroism Spectral Analysis

MP–aldehyde–gingerol mixtures were equilibrated for 40 min at 25 ± 1 °C, diluted 15-fold, and analyzed from 200 to 269 nm at 297 K using a J-1500 circular dichroism spectropolarimeter (JASCO Corporation, Tokyo, Japan). Spectra were normalized as mean residue ellipticity and evaluated at the α-helix-associated bands near 208 and 222 nm. Far-UV CD is widely used to monitor protein secondary-structure-related spectral features, and comparable MP–aldehyde studies have used changes in this region to assess treatment-related conformational responses [17,33]. In the present study, band position, shape, and magnitude were compared among treatments. Accordingly, CD provided direct spectroscopic evidence of conformational response, although exact secondary-structure fractions were not assigned because validated spectral deconvolution and fit-quality assessment were not available.

### 2.8. Molecular Docking

The three-dimensional MYH7 structure associated with UniProt accession F1QZW1 was obtained through UniProt and inspected in PyMOL 2.3.0 (Schrödinger, LLC, New York, NY, USA). The UniProt-distributed predicted structure was treated as a model rather than an experimentally resolved structure; local confidence and unresolved flexibility therefore remain relevant to pose interpretation [34]. Three-dimensional ligand structures for 6-, 8-, and 10-gingerol, T2D, DDE, and E2D were obtained from PubChem and minimized in Open Babel 3.1.1 (Open Babel Development Team; open-source software) using the MMFF94 force field. AutoDockTools 1.5.6 (Center for Computational Structural Biology, The Scripps Research Institute, La Jolla, CA, USA) was used to add hydrogens to the receptor, add hydrogens and assign rotatable bonds to the ligands, and generate PDBQT files. Semi-flexible docking was performed with AutoDock Vina 1.2.5 (Center for Computational Structural Biology, The Scripps Research Institute, La Jolla, CA, USA) using a search grid centered at x = 74, y = −25, and z = 173, with dimensions of 26 × 24 × 26 Å and exhaustiveness = 25. The best-ranked poses were visualized in PyMOL 2.3.0 and Discovery Studio 2019 (Dassault Systèmes BIOVIA, San Diego, CA, USA). A dual-ligand docking calculation placed 10-gingerol and DDE in the same receptor search region to examine whether the two ligands occupied overlapping space. Docking scores were used for within-protocol ranking and were not treated as experimental free energies.

### 2.9. Molecular Dynamics and MM/GBSA Analysis

Binary MYH7–10-gingerol and MYH7–DDE complexes were simulated using GROMACS 2024.4 (GROMACS Development Team; open-source software). The Amber14SB and GAFF2 force fields described the protein and ligand, respectively; TIP4P water filled a periodic box extending 1.2 nm from the solute. Long-range electrostatics were treated by particle-mesh Ewald, and Na^+^/Cl^−^ ions were placed by a Monte Carlo procedure to neutralize each system. Energy minimization used up to 50,000 steepest-descent steps and stopped when the maximum force fell below 1000 kJ mol^−1^ nm^−1^. Each system then underwent 100 ps NVT equilibration at 310 K and 100 ps NPT equilibration at 310 K and 1 bar, followed by an unrestrained 100 ns production run with a 2 fs time step; coordinates were stored every 10 ps. Root-mean-square deviation (RMSD), residue fluctuation (RMSF), solvent-accessible surface area (SASA), protein–ligand hydrogen bonds, representative structures, and free-energy landscapes were analyzed. MM/GBSA estimates were calculated from the trajectories and interpreted as comparative scores rather than absolute experimental affinities [35].

### 2.10. Statistical Analysis

Wet-laboratory experiments were performed in triplicate and are reported as mean ± standard deviation. One-way analysis of variance followed by Duncan’s multiple range test was conducted in DPS 7.5 (Ruifeng Information Technology, Hangzhou, China). Different lowercase letters indicate significant differences at *p* < 0.05. This analysis plan was applied consistently to all wet-laboratory treatment comparisons. Origin 2019 (OriginLab Corporation, Northampton, MA, USA) was used for plotting. Docking and MD outputs were analyzed as computational trajectories and were not included in the triplicate wet-laboratory ANOVA.

## 3. Results and Discussion

### 3.1. Effects of Gingerol Homologues on the Apparent Retention of Aldehydes

**Figure 1:**
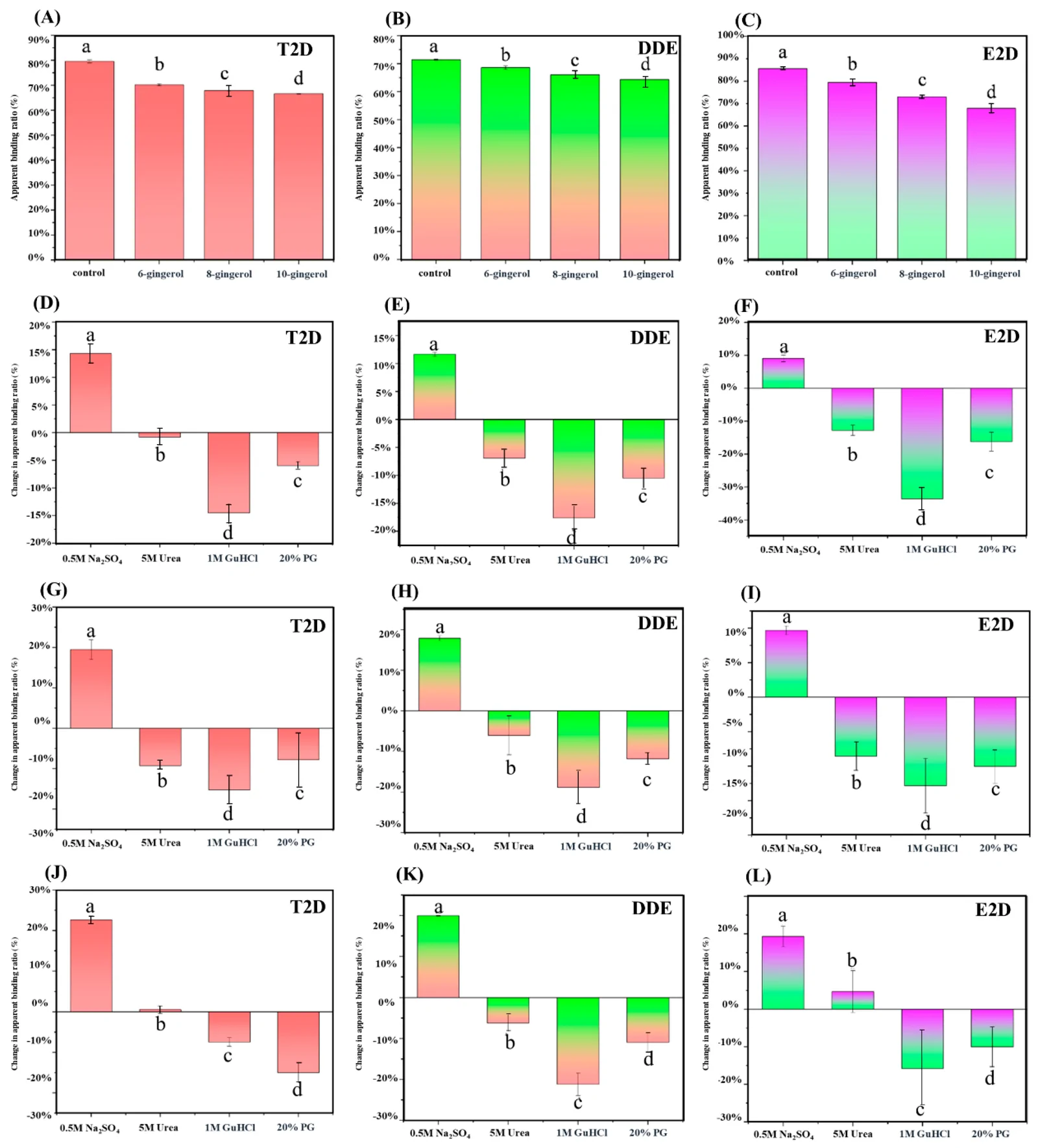
Effects of gingerol homologues and interaction disruptors on the apparent binding of aldehydes by mackerel myofibrillar protein. Columns correspond to T2D ((**A**,**D**,**G**,**J**); red/pink), DDE ((**B**,**E**,**H**,**K**); green-to-pink gradient), and E2D ((**C**,**F**,**I**,**L**); purple-to-green gradient); the gradients identify aldehyde series and do not encode a numerical scale. (**A**–**C**) Apparent binding ratios without gingerol (control) or with 6-, 8-, or 10-gingerol at 125 μmol/g protein. (**D**–**F**), (**G**–**I**), and (**J**–**L**) show changes caused by disruptors in systems containing 6-, 8-, and 10-gingerol, respectively, relative to the corresponding system without disruptor. Positive and negative bars indicate increased and decreased apparent binding, respectively; *y*-axis ranges differ among panels. Disruptors, from left to right, were 0.5 mol/L Na_2_SO_4_, 5 mol/L urea, 1 mol/L GuHCl, and 20% (*v*/*v*) PG. Bars and error bars represent mean and SD (n = 3), respectively. Different lowercase letters within a panel indicate *p* < 0.05 by the analysis described in Section 2.10.

In the absence of gingerol, MP showed apparent binding ratios of approximately 81% for T2D, 72% for DDE, and 85% for E2D (Figure 1A–C). Addition of each gingerol lowered the corresponding value, and 10-gingerol produced the lowest value within each aldehyde series. With 10-gingerol, T2D, DDE, and E2D binding fell to approximately 68%, 65%, and 68%, representing decreases of about 13, 7, and 17 percentage points, respectively (*p* < 0.05). Relative to the corresponding gingerol-free values, these decreases were approximately 16.0%, 9.7%, and 20.0%, calculated from the rounded binding ratios as (control − treatment)/control × 100. These descriptive relative decreases are distinct from percentage-point changes and from standardized statistical effect sizes. The larger response of E2D and smaller response of DDE suggest that the shared C10 backbone did not fully account for the observed response.

This overall direction agrees with the idea that phenolic–protein association can reduce the number or accessibility of MP sites available to odorants [19]. It also extends recent evidence that spice flavor compounds and structure-modifying treatments can remodel MP and regulate its flavor-binding behavior [23,36]. The side chains of 6-, 8-, and 10-gingerol become progressively longer and more hydrophobic, which can favor association with protein hydrophobic regions. However, the present result is best stated as a trend in apparent headspace-derived retention, not as proof that longer gingerols have a universally larger equilibrium binding constant. Studies of fishy compounds have shown that pH and protein conformation can change both the magnitude and direction of MP–aldehyde responses [37,38], and thermally induced unfolding or degradation can likewise expose or remove aroma-binding regions [39].

The three test aldehydes also connect protein binding to lipid-oxidation chemistry. Lipid-derived aldehydes can participate in fish MP lipoxidation [40]. DDE can act as a critical intermediate during linoleic-acid thermal oxidation [41]. T2D can itself undergo further oxidation through multiple radical and peroxide pathways [42]. Their different unsaturation and epoxide features therefore provide chemically grounded contrasts even though all three molecules contain ten carbon atoms. Specifically, T2D contains one C2=C3 double bond, DDE contains conjugated C2=C3 and C4=C5 double bonds, and E2D contains a C2=C3 double bond and a 4,5-epoxide group. These differences can alter molecular shape and the distribution of polar and hydrophobic contacts with MP. Figure 1 responses are therefore consistent with structure-dependent association and partitioning, but they cannot identify a particular binding residue or establish covalent adduct formation.

#### Analysis of Non-Covalent Interactions Using Chemical Disruptors

Na_2_SO_4_ increased the apparent binding ratio across the gingerol–aldehyde systems by 9.05–22.64% (Figure 1D–L). The largest increase occurred for T2D with 10-gingerol (22.64%), whereas the smallest occurred for E2D with 6-gingerol (9.05%). This salt-out response is consistent with enhanced hydrophobic association or altered aldehyde partitioning at high sulfate concentration. Urea produced changes from −12.87% to +2.33%, with most systems decreasing. GuHCl decreased all systems by 7.43–33.55%; the E2D–6-gingerol combination was most sensitive. PG decreased binding by 5.03–16.22%.

Taken together, the pattern is compatible with an important role for hydrophobic association, accompanied by other conformation- and solvent-sensitive non-covalent interactions. Similar system dependence has been reported for fish MP–odorant binding under changing ionic strength and pH [37] and for aldehyde binding after glycation or heat-induced structural change [43,44]. The result does not identify a percentage contribution for any single force. Na_2_SO_4_, urea, GuHCl, and PG can simultaneously change protein folding, aggregation, hydration, and gas–liquid partitioning. The combined pattern therefore suggests that MP structural integrity and solvent-sensitive non-covalent association jointly shape the measured retention.

### 3.2. UV–Visible Spectral Analysis of MP–Aldehyde Mixtures in the Presence of Gingerols

**Figure 2:**
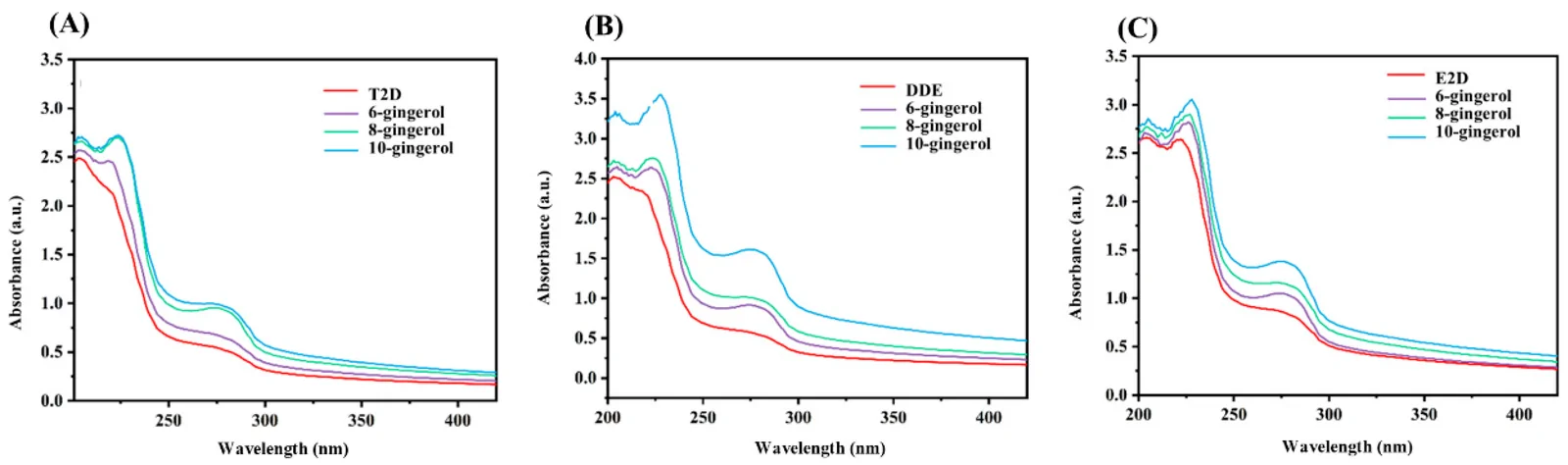
UV–visible absorption spectra of MP–aldehyde systems containing no gingerol (MP) or 6-, 8-, or 10-gingerol. (**A**) T2D, (**B**) DDE, and (**C**) E2D.

All MP–aldehyde systems absorbed strongly from 200 to 300 nm, with a prominent region near 220–230 nm and a shoulder around 270–290 nm (Figure 2). Adding gingerols increased the apparent absorbance in the order 10-gingerol > 8-gingerol > 6-gingerol > control. The response is consistent with superposition of the gingerol chromophore, altered aromatic-residue environments, and scattering from protein association or aggregation. It is not, by itself, evidence that 10-gingerol had the largest protein-binding constant. Polyphenol-induced aggregation and changes in MP surface properties have been observed in other muscle-protein systems [45,46], while gingerol–protein binding can also produce conformational compaction [24]. Thus, the UV result supports a changed optical and conformational environment but does not isolate the underlying molecular event.

### 3.3. Intrinsic and Synchronous Fluorescence Spectra of Gingerol–MP–Aldehyde Systems

**Figure 3:**
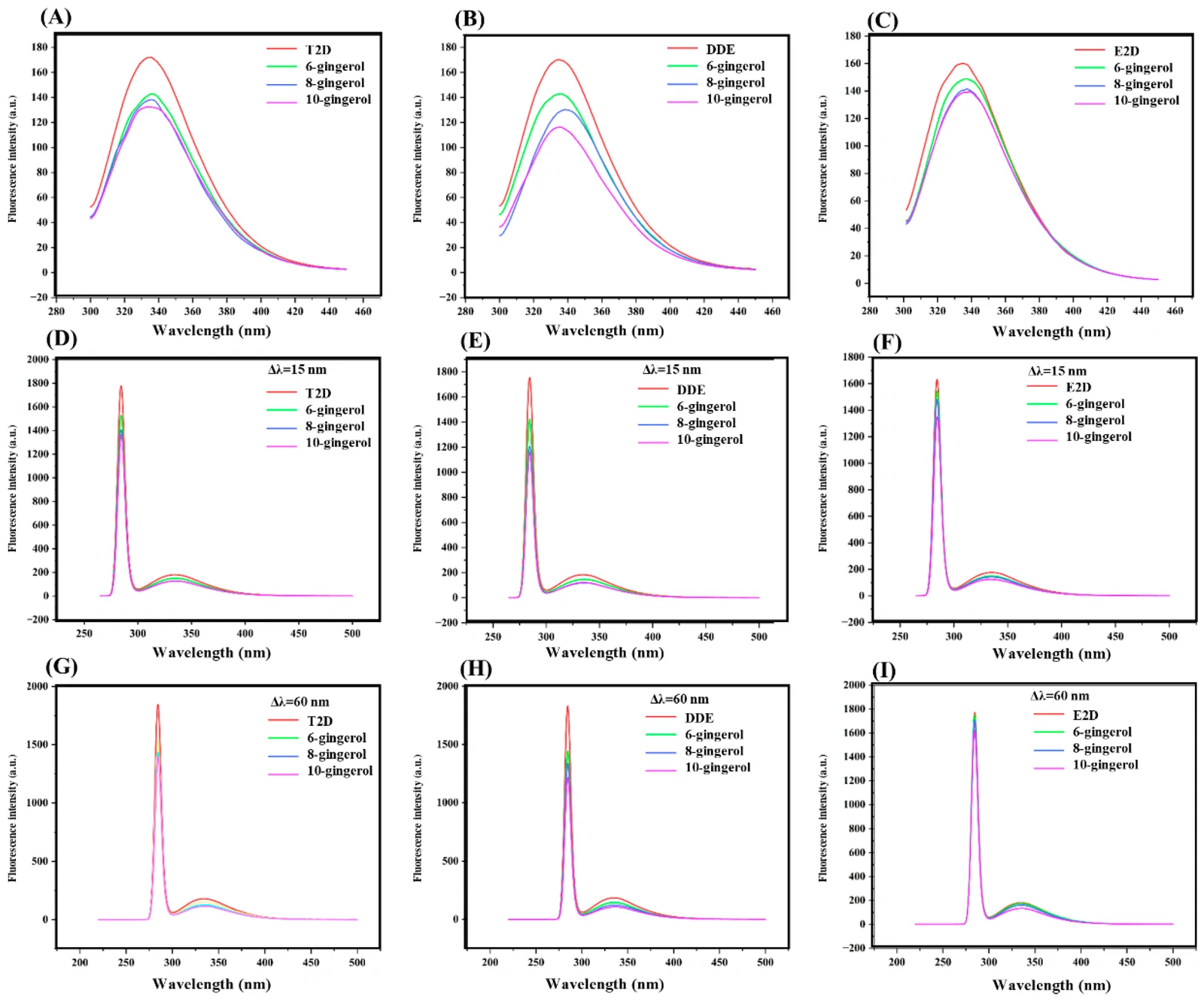
Intrinsic and synchronous fluorescence spectra of MP–aldehyde systems containing no gingerol (MP) or 6-, 8-, or 10-gingerol. (**A**–**C**) Intrinsic emission spectra of T2D, DDE, and E2D systems, respectively. (**D**–**F**) Synchronous spectra at Δλ = 15 nm. (**G**–**I**) Synchronous spectra at Δλ = 60 nm.

The intrinsic fluorescence maxima of the T2D, DDE, and E2D systems remained near 330–340 nm (Figure 3A–C). Gingerol addition reduced fluorescence intensity, generally in the order control > 6-gingerol > 8-gingerol > 10-gingerol. DDE showed the largest decrease, followed by T2D, whereas E2D changed less. The absence of a clear emission-maximum shift means that the spectra primarily demonstrate an intensity response; they do not establish a specific polarity change around aromatic residues.

Synchronous fluorescence resolved the same structure dependence. At Δλ = 15 nm, the control intensities for T2D, DDE, and E2D were 1777, 1756, and 1633 a.u. Relative quenching by 6-, 8-, and 10-gingerol was 14.1%, 20.9%, and 23.2% for T2D; 19.0%, 31.2%, and 34.2% for DDE; and 5.3%, 9.2%, and 17.5% for E2D. At Δλ = 60 nm, the corresponding values were 17.0%, 22.2%, and 24.3% for T2D; 21.7%, 26.9%, and 33.6% for DDE; and 1.3%, 3.7%, and 8.4% for E2D. DDE was therefore most responsive in both Tyr- and Trp-weighted spectra. For 10-gingerol, the DDE response exceeded the E2D response by 16.7 percentage points at Δλ = 15 nm and 25.2 percentage points at Δλ = 60 nm. This contrasts with the larger decrease in apparent retention for E2D in Figure 1, showing that local fluorescence attenuation and bulk headspace redistribution do not provide interchangeable measures of protein–aldehyde association.

These observations are compatible with gingerol association near aromatic-residue environments or with gingerol-induced protein rearrangement. Comparable fluorescence and conformational responses have been described for MPs interacting with aldehydes, ketones, alcohols, furans, and pyrazines [16,47,48,49,50]. However, no numerical inner-filter correction was applied. Fluorescence loss therefore cannot distinguish static complex formation, collisional quenching, energy transfer, or inner-filter attenuation. The spectra are interpreted only as treatment-dependent apparent quenching and not as evidence for a specific quenching mechanism or binding constant.

### 3.4. Circular Dichroism Spectral Analysis of MP–Aldehyde Mixtures in the Presence of Gingerols

**Figure 4:**
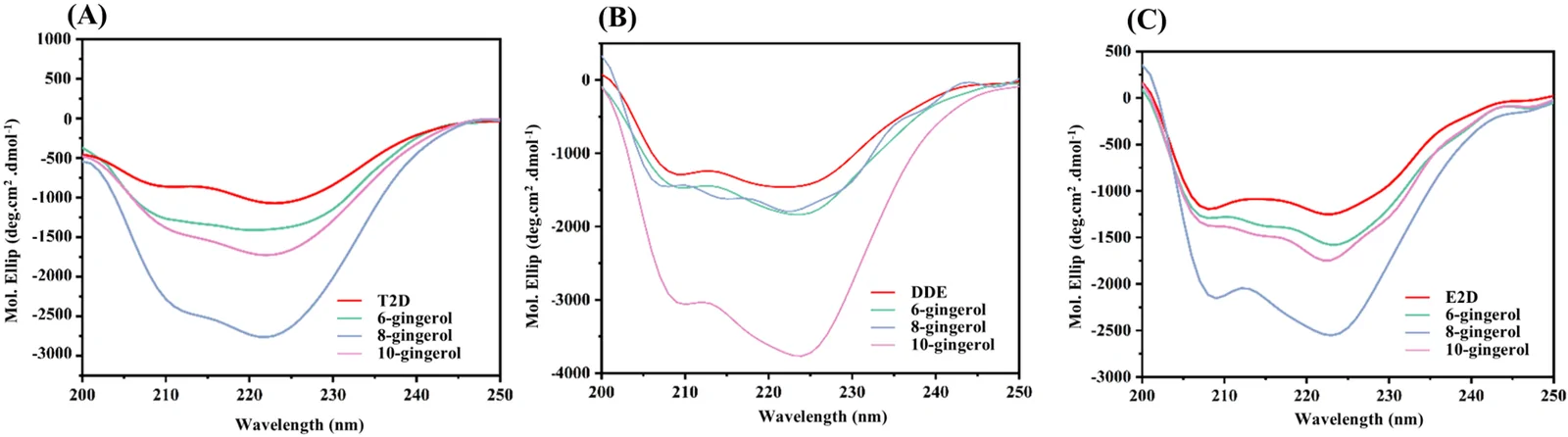
Far-UV circular dichroism spectra of MP–aldehyde systems containing no gingerol (MP) or 6-, 8-, or 10-gingerol. (**A**) T2D, (**B**) DDE, and (**C**) E2D. Mean residue ellipticity is reported in deg·cm^2^·dmol^−1^.

Each system retained two negative bands near 208 and 222 nm (Figure 4), indicating that α-helix-associated spectral features remained evident after treatment. Gingerols generally increased the magnitude of negative ellipticity relative to the aldehyde–MP control, but the homologue ranking depended on the aldehyde. In T2D and E2D systems, the approximate order was 8-gingerol > 10-gingerol > 6-gingerol > control; in the DDE system, it was 10-gingerol > 8-gingerol ≈ 6-gingerol > control. These treatment-dependent changes in mean residue ellipticity provide evidence that the secondary-structure-related spectral features of MP were altered while the characteristic α-helical signature was retained.

The non-monotonic pattern suggests that the conformational response reflects the combined geometry of gingerol, aldehyde, and MP rather than hydrophobicity in isolation. Ultrasound-induced site exposure can increase MP binding to spice compounds [36], different natural phenolics can either unfold or preserve helical features depending on their substituents [46], and anthocyanin-induced changes can modulate fishy-compound binding in an environment-dependent manner [51]. The CD response was therefore interpreted together with the interaction-disruptor, fluorescence, docking, and MD results rather than as the sole mechanistic evidence. Although the spectra do not yield exact secondary-structure percentages without validated deconvolution, they support a comparative interpretation of treatment-related MP conformational change.

### 3.5. Molecular Docking Analysis of Gingerols and Aldehydes with MYH7

**Figure 5:**
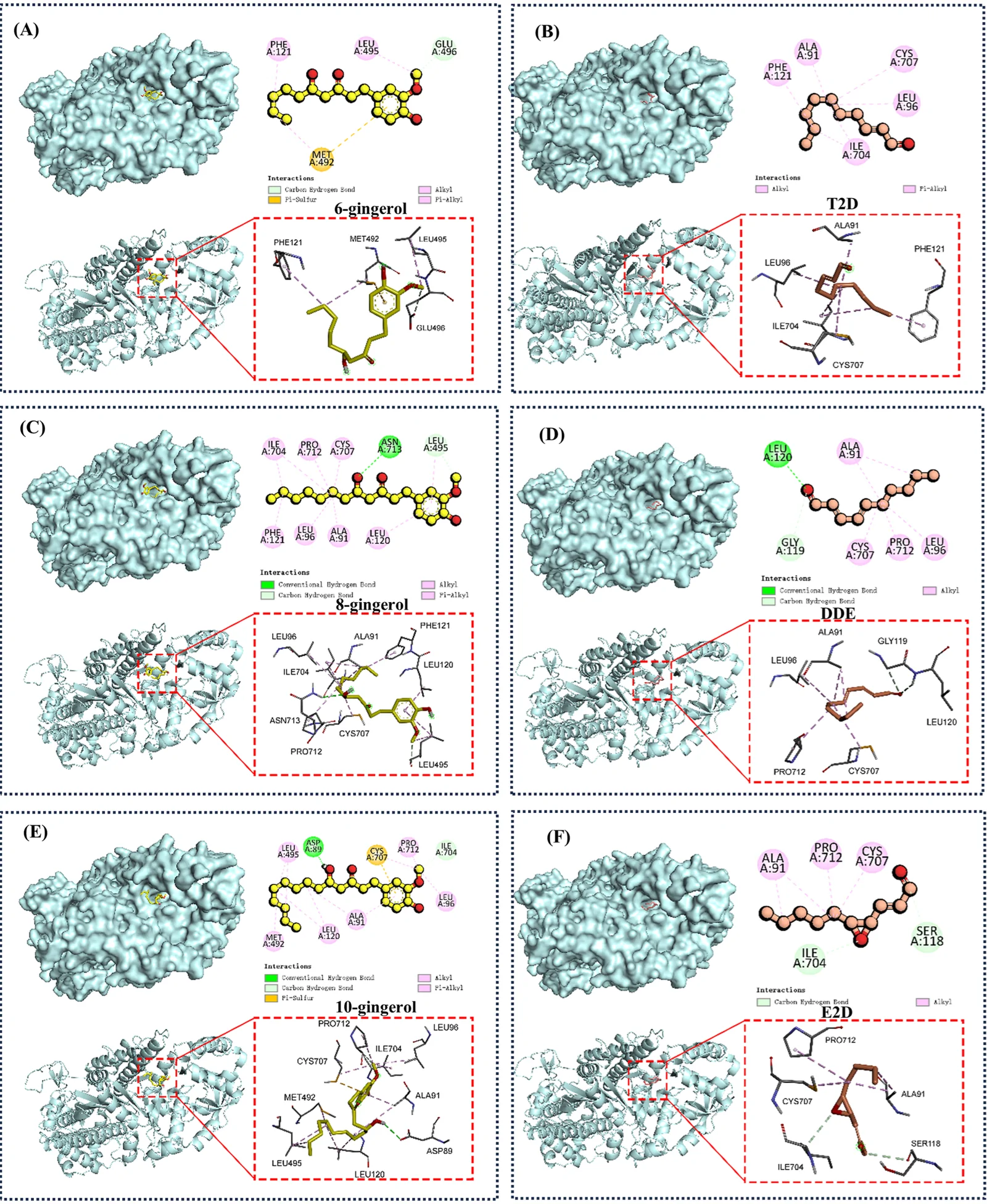
Predicted binding poses and two-dimensional interaction maps for gingerols and aldehydes in MYH7. (**A**) 6-Gingerol, (**B**) T2D, (**C**) 8-gingerol, (**D**) DDE, (**E**) 10-gingerol, and (**F**) E2D. MYH7 is shown in light cyan; the gingerols and aldehydes are shown predominantly in yellow and brown, respectively. Red dashed boxes and connectors indicate the enlarged binding regions. Colored dashed lines represent the interaction types defined in the corresponding embedded legends.

All six ligands returned negative Vina scores in the selected MYH7 pocket (Table 1; Figure 5). The scores for 6-, 8-, and 10-gingerol were −4.741, −5.810, and −5.147 kcal/mol, respectively, compared with −4.475, −4.455, and −4.537 kcal/mol for T2D, DDE, and E2D. Within this protocol, gingerols ranked more favorably than the aldehydes, but 8-gingerol—not 10-gingerol—had the most negative score. The 8-gingerol score was 0.663 kcal/mol more favorable than that of 10-gingerol, whereas the lowest experimental retention was obtained with 10-gingerol. The docking result is compatible with pocket competition but does not support a simple monotonic chain-length explanation.

Gingerols contacted a recurring cluster around ALA91, LEU96, LEU120, PHE121, MET492, LEU495, ILE704, CYS707, PRO712, and neighboring polar residues. The aldehydes occupied an overlapping hydrophobic region. The 8- and 10-gingerol poses showed broader predicted contact networks than the aldehydes, whereas 6-gingerol had a more limited network. These residues are broadly consistent with a myosin-head binding region identified for off-odor compounds [15] and with hydrophobic pockets reported in other MP–flavor docking systems [50]. Because the receptor was a predicted, partially rigid structure, the poses are best viewed as spatial hypotheses that connect the experimental trend to candidate residues.

#### Dual-Ligand Docking Analysis of 10-Gingerol and DDE in the MYH7 Binding Pocket

**Figure 6:**
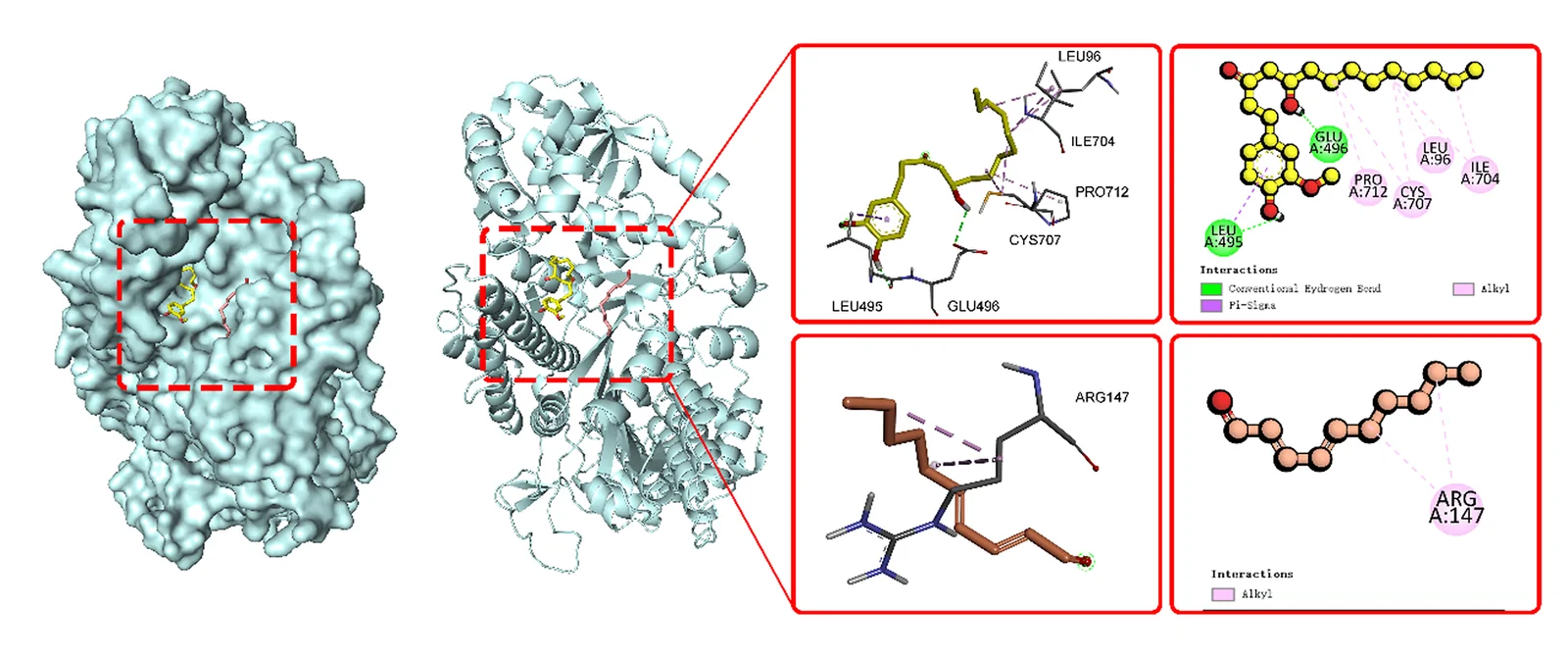
Dual-ligand docking model of 10-gingerol and DDE in MYH7. 10-Gingerol occupies the deeper part of the selected pocket, whereas DDE is positioned closer to the pocket rim. MYH7 is shown in light cyan, 10-gingerol in yellow, and DDE in brown. Red dashed boxes identify the selected binding region, while red outlines and connectors indicate the enlarged views. The colors used for molecular interactions are defined in the embedded legends.

Dual-ligand docking placed 10-gingerol deeper in the pocket and DDE closer to the rim (Figure 6). The gingerol pose formed predicted hydrogen bonds with LEU495 and GLU496, a π–σ contact with LEU495, and hydrophobic contacts involving LEU96, ILE704, CYS707, and PRO712. DDE retained only weak rim contacts around ARG147 in the dual-ligand pose. This arrangement is compatible with steric occupancy and site-access competition. It does not prove displacement in solution, but it is consistent with the lower apparent DDE retention observed when gingerol was present.

### 3.6. Comparative Molecular Dynamics and MM/GBSA Analysis of MYH7–10-Gingerol and MYH7–DDE Complexes

**Figure 7:**
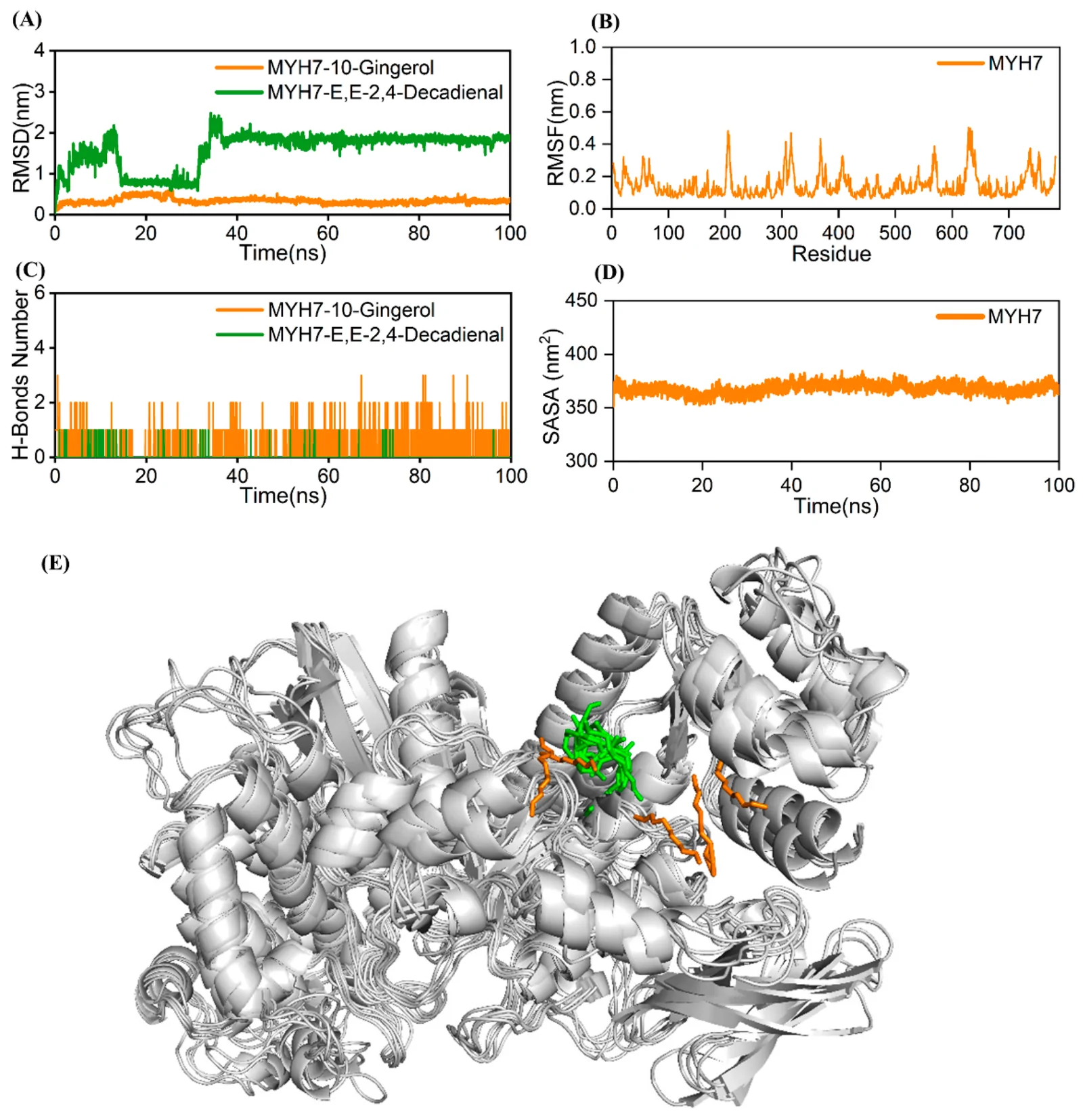
Molecular dynamics analysis of the MYH7–10-gingerol and MYH7–DDE complexes over 100 ns. (**A**) Complex RMSD, (**B**) receptor RMSF, (**C**) protein–ligand hydrogen-bond count, (**D**) receptor SASA, and (**E**) representative superposition of trajectory structures. In panels (**A**,**C**), orange and green represent the MYH7–10-gingerol and MYH7–DDE systems, respectively. The orange traces in panels (**B**,**D**) represent MYH7. In panel (**E**), the superposed MYH7 structures are shown in gray, whereas 10-gingerol and DDE are shown in orange and green, respectively.

The MYH7–10-gingerol complex maintained an RMSD near 0.3 nm and remained below 1 nm throughout the selected 100 ns simulation (Figure 7A). The MYH7–DDE trajectory fluctuated more during the first 40 ns and later approached approximately 2 nm. This larger value is consistent with substantial pose or domain rearrangement, although its magnitude also depends on the atom selection and alignment convention used for RMSD. Residue fluctuations remained mostly below 1 nm in both trajectories (Figure 7B). The 10-gingerol trajectory intermittently formed one to two protein–ligand hydrogen bonds and occasionally three, whereas DDE did not retain a persistent hydrogen bond (Figure 7C). SASA varied modestly (Figure 7D), and trajectory snapshots showed 10-gingerol remaining in the pocket while DDE moved toward solvent (Figure 7E).

**Figure 8:**
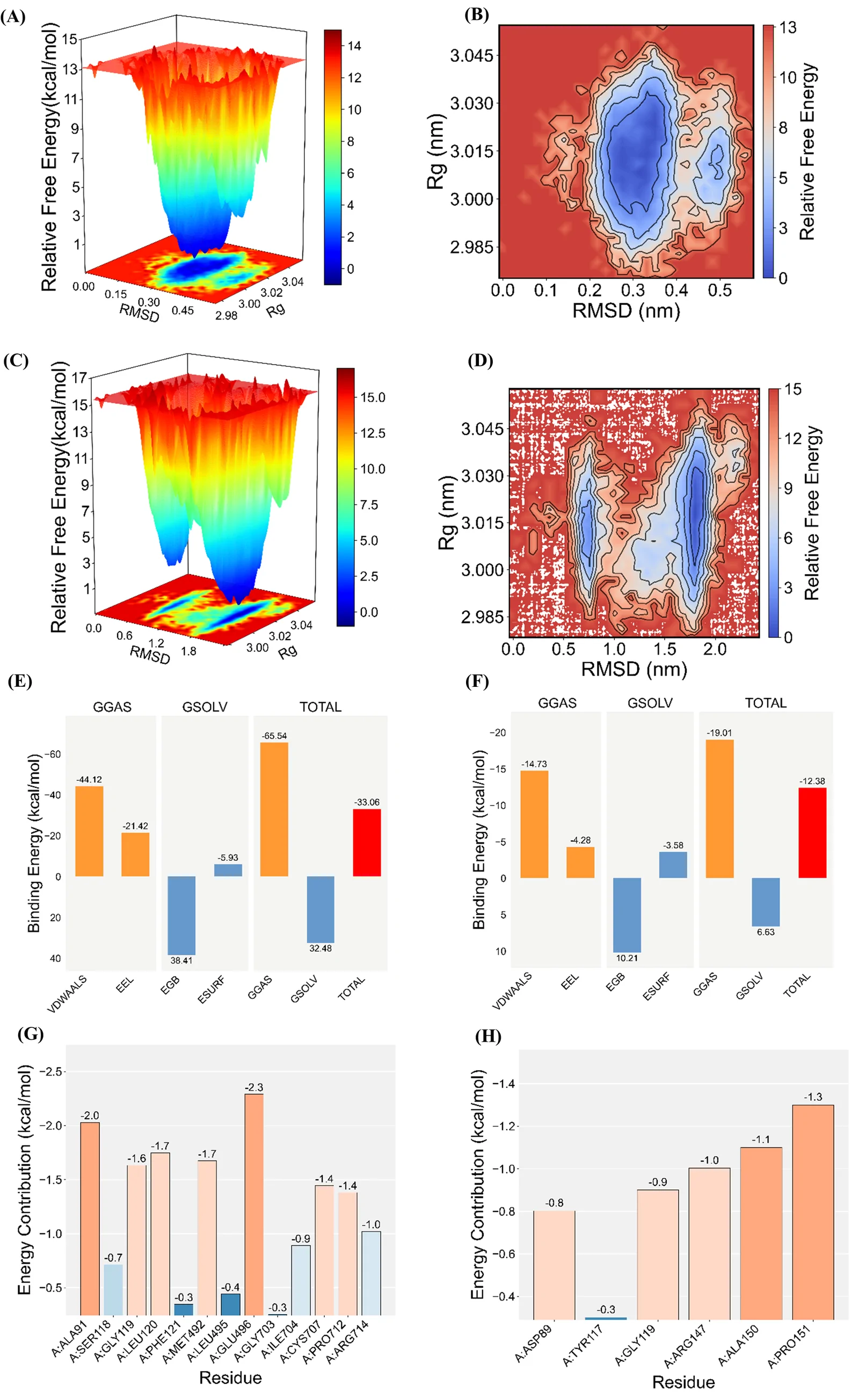
Free-energy landscape and MM/GBSA analysis of MYH7–10-gingerol and MYH7–DDE complexes. (**A**,**B**) Three-dimensional and contour free-energy landscapes for MYH7–10-gingerol; (**C**,**D**) corresponding landscapes for MYH7–DDE; (**E**,**F**) MM/GBSA energy-component summaries for 10-gingerol and DDE, respectively; and (**G**,**H**) corresponding per-residue energy contributions.

The free-energy landscape of MYH7–10-gingerol contained one dominant low-energy basin, whereas the DDE complex sampled two more separated basins (Figure 8A–D). Comparative MM/GBSA estimates were −33.06 kcal/mol for 10-gingerol and −12.38 kcal/mol for DDE (Figure 8E,F). The two MM/GBSA estimates differed by 20.68 kcal/mol in favor of 10-gingerol within the selected trajectories; this difference does not quantify an experimental displacement free energy. GLU496, ALA91, LEU120, and MET492 contributed approximately −2.3, −2.0, −1.7, and −1.7 kcal/mol to the 10-gingerol estimate, respectively (Figure 8G). The DDE decomposition was weaker, with the largest reported contributions near PRO151 and ALA150 (Figure 8H). Across these model-dependent descriptors, 10-gingerol showed a more persistent selected pose than DDE. Similar multi-technique studies have used MD to distinguish stable and mobile MP–ligand poses [16,49,52], but the numerical MM/GBSA values remain comparative estimates rather than experimental free energies.

### 3.7. Integrated Analysis and the Three-Layer Competition–Partitioning Model

The combined data support a three-layer working model. At the matrix layer, gingerols and disruptors change the distribution of aldehydes among liquid, protein-associated, and headspace states; HS-SPME records the net result. At the protein layer, gingerol association changes aggregation, hydration, and the accessibility of MP binding regions, as indicated by UV, fluorescence, and CD responses. At the pocket layer, gingerols and aldehydes can occupy overlapping MYH7 contacts; in the two modeled trajectories, 10-gingerol remained associated with the selected pocket more consistently than DDE. This model provides a framework for interpreting why the headspace trend appears broadly chain-length-related while docking and CD show non-monotonic details.

The model also clarifies how altered protein retention may affect flavor management in fish products. During preliminary method development, an exploratory sensory pretest of gingerol-treated mackerel samples indicated a perceptible improvement in the aldehydic/fishy odor profile and supported the feasibility of the research direction. Because this pretest was used to inform experimental design rather than as a formal blinded and replicated sensory trial, it is not treated as confirmatory study data. The present experiments were therefore designed to clarify the underlying mechanism at the matrix, protein, and molecular levels. Reduced protein retention can increase aldehyde release into the headspace, which is useful when the released compounds are subsequently removed, diluted, transformed, or sensorially balanced. Related fish-protein systems have shown phenolic promotion of fishy-odorant release [19]. In a closed product, however, greater release could transiently increase headspace odor intensity. Application during curing or subsequent processing therefore requires a removal pathway for released aldehydes, together with control of ongoing oxidation; neither removal efficiency nor sensory benefit was quantified in the present MP model.

Three observations sharpen the mechanistic interpretation. First, 10-gingerol produced the lowest apparent aldehyde retention, yet 8-gingerol had the most favorable docking score. Second, 8-gingerol produced the largest CD response in T2D and E2D systems, whereas 10-gingerol dominated in DDE. Third, E2D showed the largest decrease in apparent binding but the smallest fluorescence response. These contrasts indicate that aldehyde structure, pocket geometry, and matrix partitioning modulate the effect of gingerol hydrophobicity. They also prevent a local observation—such as one docking score—from being generalized into a universal relationship.

Further validation should measure residual aldehyde content, headspace release, and blinded sensory responses together in a real cured-mackerel matrix. Comparisons across gingerol dose and processing conditions should also consider desirable aroma retention and ginger-derived sensory notes. This would establish whether the reduced apparent MP retention observed here translates into a net improvement in product flavor under practical conditions.

## 4. Conclusions

Gingerol homologues reduced the apparent retention of three C10 aldehydes by mackerel MP, with 10-gingerol producing the lowest values under the tested conditions. The combined evidence supports a working model in which matrix partitioning, protein-site accessibility, and pocket occupancy jointly influence retention.

The findings provide a mechanistic basis for evaluating gingerol addition during curing to limit the persistence of aldehydic off-notes, provided that released aldehydes are subsequently removed or sensorially balanced. An exploratory sensory pretest supported the feasibility of this application, while formal sensory profiling in a real mackerel matrix remains desirable for quantitative confirmation. Other limitations include the absence of numerical inner-filter correction, exact CD secondary-structure fractions, and replicate MD trajectories. The results therefore establish a molecular basis for a practical release-modulation strategy rather than claiming complete deodorization.

## Figures and Tables

**Table 1:** Docking scores and representative MYH7 contacts for gingerols and aldehydes.

Representative Predicted Contacts	Vina Score (kcal/mol)	Ligand
GLU496, MET492, PHE121, LEU495	−4.741	6-Gingerol
ASN713, LEU495, ALA91, LEU96, LEU120, PHE121, ILE704, CYS707, PRO712	−5.810	8-Gingerol
ASP89, ILE704, CYS707, ALA91, LEU96, LEU120, MET492, LEU495, PRO712	−5.147	10-Gingerol
ALA91, LEU96, PHE121, ILE704, CYS707	−4.475	T2D
LEU120, GLY119, ALA91, LEU96, CYS707, PRO712	−4.455	DDE
SER118, ILE704, ALA91, CYS707, PRO712	−4.537	E2D

## Data Availability

The original contributions presented in the study are included in the article, further inquiries can be directed to the corresponding authors.
